# Low-Quality Video Target Detection Based on EEG Signal Using Eye Movement Alignment

**DOI:** 10.34133/cbsystems.0121

**Published:** 2024-07-04

**Authors:** Jianting Shi, Luzheng Bi, Xinbo Xu, Aberham Genetu Feleke, Weijie Fei

**Affiliations:** School of Mechanical Engineering, Beijing Institute of Technology, Beijing 100081, China.

## Abstract

The target detection based on electroencephalogram (EEG) signals is a new target detection method. This method recognizes the target by decoding the specific neural response when an operator observes the target, which has important theoretical and application values. This paper focuses on the EEG detection of low-quality video targets, which breaks through the limitation of previous target detection based on EEG signals only for high-quality video targets. We first design an experimental paradigm for EEG-based low-quality video target detection and propose an epoch extraction method based on eye movement signals to solve the asynchronous problem faced by low-quality video target detection. Then, the neural representation in the process of operator recognition is analyzed based on the time domain, frequency domain, and source space domain, respectively. We design the time-frequency features based on continuous wavelet transform according to the neural representation and obtain an average decoding test accuracy of 84.56%. The research results of this paper lay the foundation for the development of a video target detection system based on EEG signals in the future.

## Introduction

In recent years, the unmanned aerial vehicle (UAV) industry has played an increasingly important role in military reconnaissance, space remote sensing, smart city, disaster monitoring, and other aspects due to its high altitude, high speed, stealth, and long-endurance characteristics. Compared with the traditional manual inspection methods, UAV detection is more flexible and convenient. Equipped with cameras and a Global Positioning System, drones can carry out autonomous patrols along specified routes and upload photos and videos in real time, providing a large amount of information. Therefore, how to detect critical targets accurately and efficiently from drone videos has become a hot research topic [1–3].

Generally, there are 2 methods to detect objects from videos: human and machine recognition. Machine vision technology has developed rapidly in recent years. The efficiency of image processing and recognition is very high. However, identifying low-quality targets remains a challenge for machine vision [4]. The “low-quality” used in this paper refers to video objects with the characteristics of camouflage, mutilation, occlusion, blur, low signal-to-noise ratio, low resolution, and so on. Due to the influence of many uncontrollable factors, such as environment and weather, the video data collected by UAVs are often characterized by many low-quality targets, which makes it difficult for machine vision to achieve the requirements of sensitivity, versatility, and reliability. In contrast, human cognitive ability is very good at discovering low-quality video targets that are difficult to be identified by machine vision [5]. However, the processing speed of human recognition is slow, and the massive amount of video data is easy to make people fatiguing and bring challenges to manual processing.

To solve the shortcomings of human recognition, many researchers have proposed using a brain–computer interface (BCI) for target recognition. BCI is a new human–computer interaction method that can directly “translate” brain activities and realize the operation of external devices [6–8]. BCI can be used to directly detect the specific neural response of humans when they find targets, namely, event-related potentials (ERPs). By analyzing and identifying the ERPs, the target can be detected. Gerson et al. [9] showed that an ERP-based target detection system can achieve an average detection accuracy of 92%, which is not significantly different from the accuracy of manual button pressing. The ERP-based method can also improve the performance decline of manual detection due to operators’ fatigue to a certain extent.

Although many researchers have applied BCIs to target detection, most focus on image-target detection [10–12], and a few focus on video-target detection [13,14]. In this paper, we focus on video-target detection based on ERPs. A video contains more information than an image and is closer to the natural condition. However, it brings new challenges. When operators watch a video, a visual search process is involved, resulting in the moment of target discovery lagging behind the moment of target appearance at random intervals, which makes it difficult to determine the moment of target recognition to accurately extract ERPs fragments. This problem is called the “asynchronous detection problem”.

To solve the asynchronous problem of video target detection, some researchers required subjects to press the key immediately after seeing the target and took the key-pressing moment as the moment when subjects discovered the target. However, manually pressing the key introduces difficulty in accurately extracting the ERP component. Some researchers applied the paradigm of rapid serial visual presentation (RSVP) to video target detection. This method cuts the video into several photo sets of 5 to 10 frames per second and presents them to the subjects. In this way, the problem of video target detection is transformed into the problem of image target detection. For example, in 2012, Weiden et al. [15] conducted a study on specific targets, requiring subjects to identify “human” or “vehicle” targets in video scenes. In 2014, Marathe et al. [16] also studied the “person” or “vehicle” targets in the video scene and proposed the sliding hierarchical discriminant component analysis classification algorithm, which reduced the classification error by 50% compared with the existing classification algorithm. However, using the RSVP paradigm to present videos brings severe limitations. Compared with real video detection, extracting key images from videos reduces information integrity and is not conducive to real-time detection. To ensure that the time when the target appears is consistent with the time when the target is recognized, the targets in the RSVP paradigm must be clear and eye-catching, which is not consistent with the actual situation.

In recent years, several other studies have used electroencephalograms (EEGs) to detect continuous video targets. Mustafa et al. [17] studied several types of artifacts (such as blurring, ghosting, and stuttering) in videos as targets and proposed a wavelet-based algorithm, which achieved 85% classification accuracy. Liu et al. [18] studied human perception sensitivity to different distortion degrees of videos. They took the P300 component caused by changes in human perception video quality as an index of human perception distortion. However, the video targets in these studies are still high-quality targets, the time of target recognition can be approximated to be the time of target appearance, and there is no asynchronous problem.

In 2020, Song et al. [19] proposed an asynchronous video target detection algorithm, which intercepted possible ERP samples by aligning the collected samples with a preset standard ERP template. However, the ERP amplitude of single trial samples and signal-to-noise ratio are low. The alignment of the samples to the ERP template is likely to fail due to noise. All the above studies used videos with clear and eye-catching targets. Once low-quality targets appeared in the videos, the longer visual search time would make the existing methods ineffective.

To address the issue mentioned above, we first design a new paradigm for low-quality video target detection, which simulates the video taken by UAVs under the influence of weather, terrain, and other factors, including low-quality targets with characteristics such as camouflage, mutilation, occlusion, blurring, and small size. Then, we propose an EEG epoch extraction method based on eye movement signals to solve the asynchronous detection problem in low-quality video target detection. To account for the neural representation of low-quality video target perception, we analyze the neural representation from the time, frequency, and source space domains. Finally, we build an EEG decoding model for low-quality video target detection based on the neural representation and demonstrate the effectiveness of our proposed method.

The contribution of this paper is 2-fold: (a) This work is the first to explore the detection of low-quality video targets based on EEG signals, breaking the limitations of only using clear and eye-catching video objects or the RSVP paradigm. (b) This paper proposes an ERP alignment method based on eye movement signals to solve the asynchronous detection problem in video target detection and develop an EEG-based detection method of low-quality video targets, which is beneficial for the practical application of this kind of BCIs.

## Methods

### Paradigm and data acquisition 

#### Participants and experimental platform

A total of 8 subjects (7 males, 1 female, mean age of 25 ± 3 y, 8 right-handed) participated in the study. All subjects were college students with normal or corrected vision. They reported no history of neurological problems and did not take any alcohol, tobacco, drugs, or caffeine-based psychotropic drugs before the experiment. All subjects signed informed consent prior to the experiment. The study followed the principles of the 2013 Declaration of Helsinki and was approved by the Beijing Institute of Technology research ethics committee.

#### Paradigm design

**Fig. 1.**
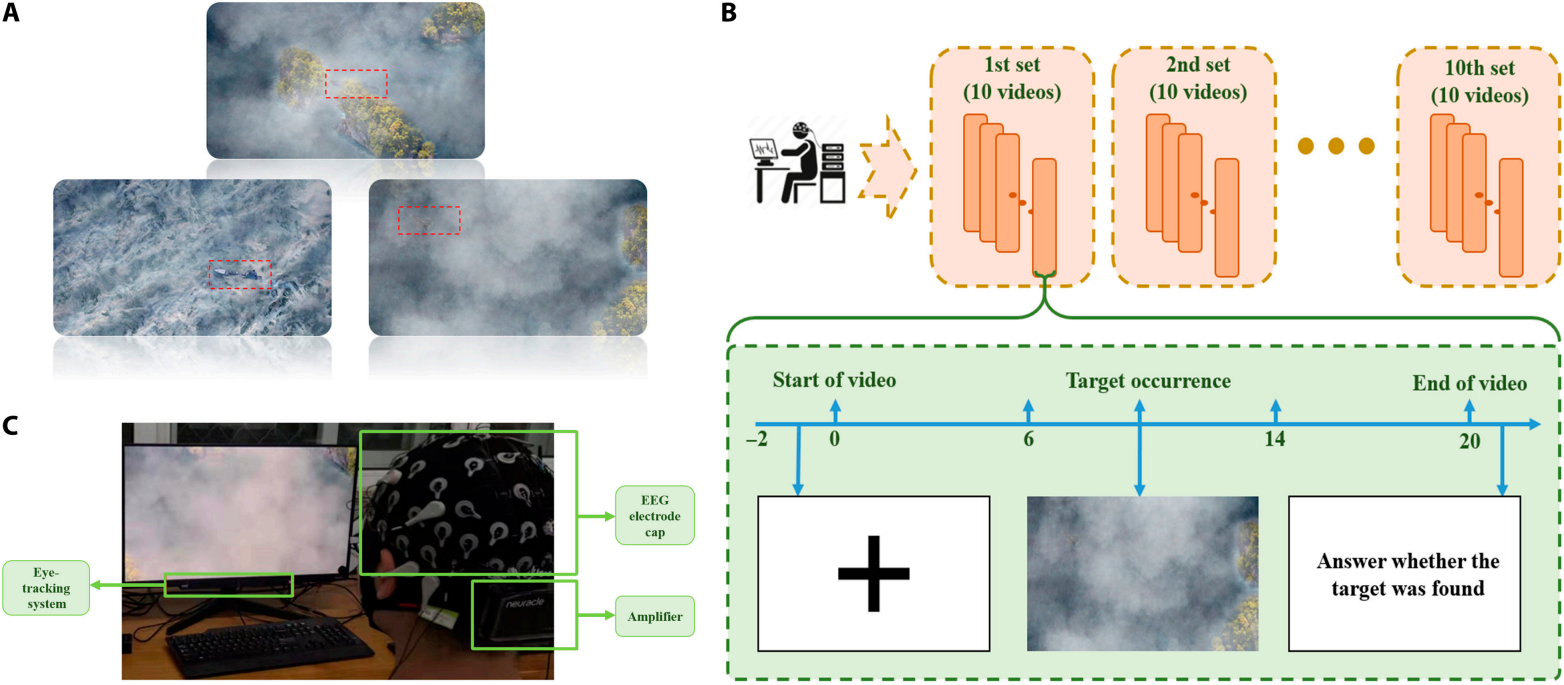
(A) An example of the screen in the experimental video. The aircraft and carrier in the red box are targets, which are hidden in the layer, wave, and island. (B) Experimental paradigm. (C) Experimental setup.

The experimental video simulated the video taken by a UAV during its reconnaissance of the sea surface. The UAV flew forward at a constant speed and captured videos with a resolution of 1,920 × 1,080 pixels, including aircraft, carriers, ships, clouds, islands, and waves. Among them, aircrafts and carriers were the target detection objects, which were hidden in the video and appeared at random positions in the video. In order to simulate the low quality of the targets caused by weather, environment, and other factors, the targets were partially obscured by clouds, sea waves, and islands, making them more difficult to be found. As shown in Fig. 1A, the task of the subject was to find the targets. A total of 240 short videos, each 20 s long, were prepared for the experiment. Half of the videos (120) contained the targets of the aircrafts or carriers, the other half did not contain the targets as nontarget videos, and each video contained at most 1 target. The subjects were asked to answer whether they found the target after the video was played.

The experimental paradigm and setup are shown in Fig. 1B and C, respectively. Subjects were asked to sit on a chair with their hands on their thighs naturally. In the formal experiment, the videos were divided into 12 sections, each containing 10 target videos. All were played at random. Each part had a 5-s relaxation period for the subjects to prepare for the experiment, and then 10 videos were shown in turn. To prevent visual overload, rest periods were provided between the 2 sections.

#### EEG and eye-tracking data acquisition

EEG acquisition was performed using the NeuSen 64-electrode Portable Wireless Electroencephalogram Amplifier (W64, Neuracle, China). According to the International 10-20 system, the forehead was grounded at AFz, and the reference is placed at CPz. The sampling rate was set to 1,000 Hz. The electrode impedance was calibrated to less than 5 kΩ.

The Tobii Pro Fusion Screen-based Eye-tracking System (Tobii, Sweden) was used to record the eye movements of the subjects during the experiment, and the sampling frequency was 60 Hz. Prior to the experiment, each participant’s eye-tracking system was calibrated. The eye movement data recorded contained the horizontal and vertical coordinates of the subject’s fixation point in the display screen for each frame.

#### Epoch extraction method based on eye movement signal

The first way of epoch extraction was aligned based on the moment of appearance of the target in the video. In this study, the Triggerbox Wireless Multi-device Synchronizer (Neuracle, China) was used to implement event labeling with an event synchronization accuracy <1 ms. The device can mark the time when the target appears in the stimulus video in the EEG signal.

**Fig. 2.**
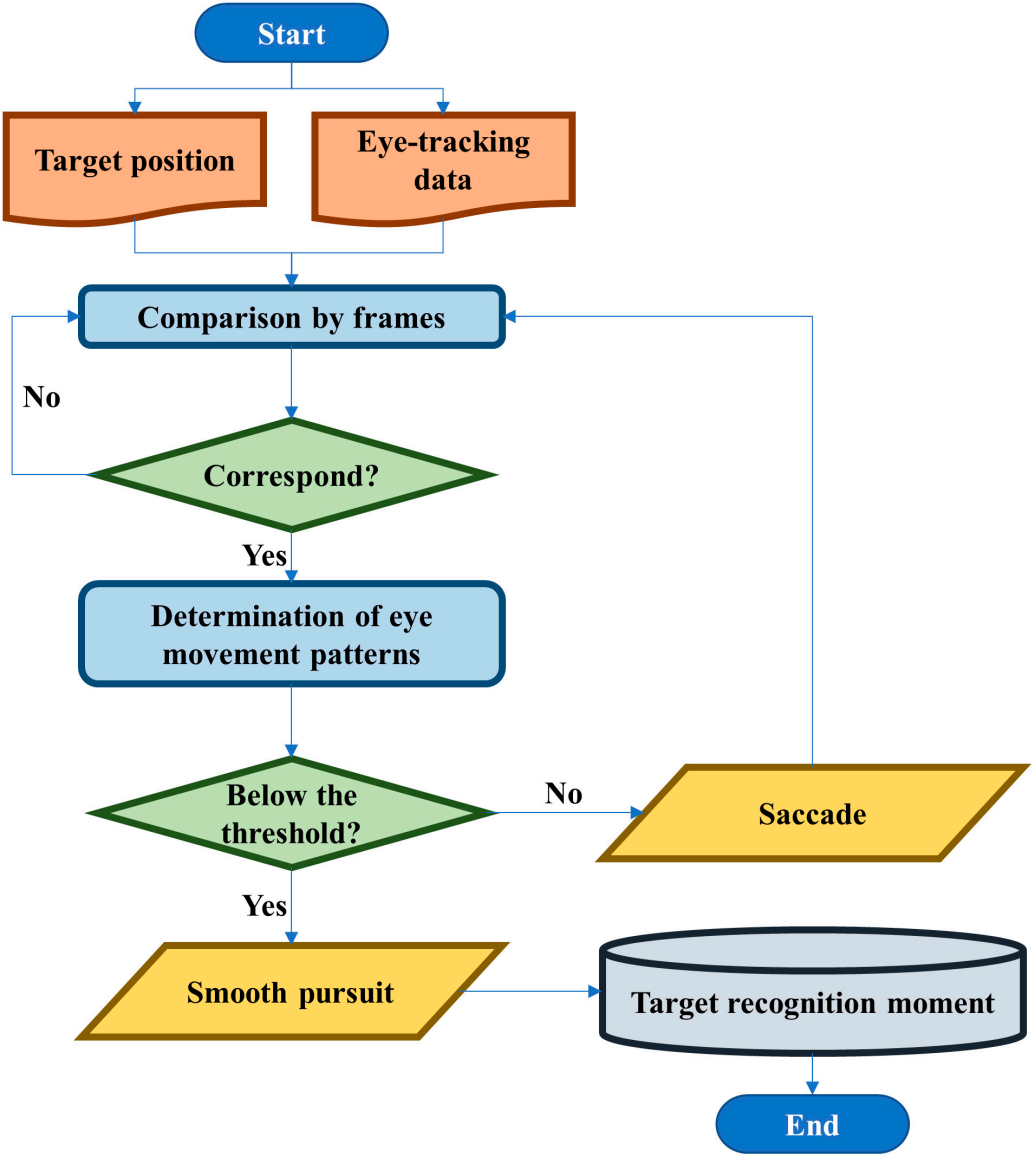
Epoch extraction method based on eye movement signal.

However, this study was an asynchronous detection problem for low-quality video target detection, and the time when subjects detected the target was randomly delayed from when the target appeared. Many studies have proved that the use of saccades and smooth pursuit in visual search tasks is closely related to search efficiency and target discovery rate [20,21], and there are existing studies supporting the relationship between different eye movement types and cognitive state of subjects [22,23]. Therefore, we proposed an ERP alignment method based on eye movement signals. The proposed method can extract ERP segments more accurately during the training phase. By using eye movement signals, it can be ensured that the extracted ERP segments are the real neural activity after the subject actually pays attention to the target, and then a training dataset that accurately reflects the subject’s brain processing can be established, so as to train a model with superior performance. The implementation steps of the proposed method are presented in Fig. 2.

For the stimulus videos, the position and time of the targets appearing in the video were prerecorded when the stimuli were made, which was the experimenter’s prior knowledge. In the course of the experiment, the subjects’ eye movements were mainly divided into 2 types. When subjects scanned and searched for the targets, the eye movement type was saccade. When the subjects found the targets, the subject’s eye movement behavior switched to smooth pursuit.

To extract EEG epochs with eye movement signals, we first compared the eye movement data sequentially with the prerecorded target locations and further determined the sampling points that coincided with the target locations. We then judged the eye movement type of the sampling point. If the sampling point was saccade, we continued to judge the next sampling point until the eye movement position coincided with the target position, and the eye movement type was smooth pursuit. This sampling point was considered as the moment when subjects recognized targets. ERP was aligned, and the EEG epoch was extracted based on this moment.

In particular, the velocity-threshold identification fixation classification algorithm was used to classify eye movement types in this study, and the speed threshold of saccade and smooth pursuit was set at 30°/s [24,25].

### Data analysis

#### EEG preprocessing

The raw EEG data were first corrected at baseline (using the first 10% of each sample as a reference and removing zero drift by subtracting the mean of the first 10%). The trials were removed with maximum absolute amplitude of EEG exceeding 200 μV, and the remaining trials were downsampled to 200 Hz. Finite impulse response band-pass filtering (0.5 to 49 Hz) was performed. After that, the independent component analysis algorithm (ICA) was used to remove eye and myoelectric artifacts. In accordance with the study of [26], we trained ICA on the data filtered to 2 to 49 Hz to maximize the performance of artifact filtering [27]. The ICLabel algorithm was used to determine each component of the independent component, among which the components labeled as “EYE” and “MUSCLE” and with a confidence greater than 90% were removed [28]. In the end, all EEGs were re-referenced to the common average.

All of the above preprocessing processes were completed through the EEGlab toolbox in MATLAB R2021b [29].

#### ERP analysis

We analyzed the EEG signals before and after target recognition in the experiment of low-quality video target recognition from the perspective of time domain. By analyzing the amplitude and latency of the ERP waveform, we compared 2 different ERP alignment methods (extracting epochs based on eye movement signals or triggerbox). The ERP components of the central, parietal (cognitive-related), and occipital (visual-related) regions (i.e., representative brain regions associated with stimuli or tasks) were analyzed in this study. To determine the electrode locations of brain activity, we mapped the brain topography from 50 to 800 ms beginning from the time of alignment to provide spatial dimension information.

#### ERSP analysis

In this part, the EEG data of the low-quality video object recognition after alignment were analyzed from the time-frequency domain, which can improve the study of the mechanism of brain activity in the process of low-quality video object recognition. Previous studies have shown that the process of target recognition also affects the EEG signal spectrum in different regions of the brain. In this paper, we applied a method of event-related spectral perturbation (ERSP) [30] to extract the time-frequency characteristics of EEG signals.

We applied a 3-cycle wavelet with an expansion factor of 0.5 to complete the time-frequency decomposition. Computations were based on frequencies ranging from 1.2 to 20 Hz with a step of 0.1 Hz. This band covered the delta, theta, and alpha bands of the EEG rhythm.

#### Source analysis

The above neural representations were all calculated for single or multiple EEG channels. In order to further enhance the interpretability of the neural representations obtained in this study and to verify and compare with existing neurological findings, the source analysis method was used to calculate the source of neural activity in the brain.

In the establishment of forward conduction model based on EEG signals, the symmetric boundary element method was used in this study, which has high geometric accuracy. To solve the dipole discharge activity at the vertex of each grid, Standardized Low Resolution brain Electromagnetic TomogrAphy (sLORETA) was used [31].

In this study, source analysis was implemented through BrainStorm software [32].

### Low-quality video target EEG decoding model

#### Dataset establishment

The specific EEG representations obtained by these analyses provided a theoretical basis for the establishment of the EEG decoding model in this section.

To construct decoding models of low-quality video objects with different features, target samples and nontarget samples needed to be obtained. We extracted 1 target sample and 1 nontarget sample from each group of experiments, and each sample window width was 1 s. Based on the epoch alignment time, we took [0, 1] s as the target sample and [−4, −3] s as the nontarget sample. We collected a total of 1,898 samples from the experimental data of 8 subjects.

In particular, we built EEG decoding models for samples obtained with 2 different epoch extraction methods to demonstrate the validity of our proposed method.

#### Feature extraction

The continuous wavelet transform was used to extract the time-frequency characteristics of EEG signals. This method inherits and develops the localization idea of short-time Fourier transform and overcomes the shortcomings of the window size not changing with frequency. It can provide a “time-frequency” window changing with frequency, which is an ideal tool for signal time-frequency analysis and processing. The continuous wavelet transform can be expressed as follows:

$${\mathrm{WT}}_{f}\left(a,b\right)=\frac{1}{\sqrt{\left|a\right|}}\int f\left(t\right)\emptyset \left(\frac{t-b}{a}\right)\mathrm{dt}$$ (1)

where *f*(*t*) is the time domain signal; $\emptyset_{a,b}\left(t\right)=\frac{1}{\sqrt{a}}\emptyset \left(\frac{t-b}{a}\right)$ is the basic displacement and scale extension of the basic wavelet ∅(*t*); and *WTf(a, b)* is the signal after wavelet transform. The scale factor *a* changes the size, shape, and structure of the basic wavelet, and the displacement factor *b* changes the location of the basic wavelet.

The preprocessed EEG signals were standardized using *z*-score, and the above continuous wavelet transform was used to calculate the EEG amplitude at different time frequencies. For the signal after wavelet transform, the power of the frequency band from 1 to 13 Hz of each channel was selected for superposition, and 200-dimensional feature vectors were obtained for each channel (each sample is 200 sample points per second). The feature vectors of 59 channels were concatenated together to be defined as time-frequency features.

#### Channel selection

We designed a channel selection method based on Max-Relevance and Min-Redundancy. Specifically, we reduced the dimensionality of the EEG data by removing redundant and irrelevant EEG channels and selected the subset of optimal channels that carries the most discriminative information Max-Relevance and Min-Redundancy.

First, we computed the Fisher score for each channel feature, which can be computed as follows:

$$J_{\text{fisher}}\left(i\right)=\frac{S_{B}^{\left(i\right)}}{S_{w}^{\left(i\right)}}=\frac{\sum_{k=1}^{c}\;\frac{n_{k}}{n}{\left({\bar{m}}_{k,i}-{\bar{m}}_{i}\right)}^{2}}{\frac{1}{n}\sum_{k=1}^{c}\;\sum_{j=1}^{n_{k}}\;{\left(x_{k,j,i}-{\bar{m}}_{k,i}\right)}^{2}}$$ (2)

where *J_fisher_*(*i*) denotes the F-score of the *i*-th feature. $S_{B}^{\left(i\right)}$ denotes the interclass variance of the *i*-th feature on the dataset. $S_{w}^{\left(i\right)}$ denotes the intraclass variance of the *i*-th feature on the dataset. ${\bar{m}}_{i}$ and ${\bar{m}}_{k,i}$ represent the average of the *i*-th feature corresponding to the entire dataset and *k*-th class, respectively. *x*_*k*, *j*, *i*_ is the value of the *i*-th feature in the *j*-th sample of class *k*. *n_k_* is the number of samples in the *k*-th class. Fisher score is an effective feature selection method. Its main idea is to take the features with the smallest intraclass distance and the largest interclass distance as the features with strong discrimination performance. We used it to filter out the best subset of features. The Fisher scores of all channels were calculated and sorted in descending order. The channel with the highest Fisher score was selected to join the feature space in order, and the accuracy was calculated by support vector machine (SVM) classifier. The channels that can increase the accuracy were kept; otherwise, they were discarded.

#### Off-line training and validation

The feature vectors after channel selection still had a high feature dimension. In order to prevent overfitting in the training process of EEG decoding model, we used principal component analysis (PCA) to compress the original feature vectors. In this study, the PCA eigenvalue contribution rate threshold was 90%, and the compressed feature vector was used as the final features for model training.

We selected 3 different several machine learning algorithms: SVM, XGBoost, and regularized linear discriminant analysis (RLDA) for offline training of the classifier. Six-fold cross validation method was used to train and test the samples of each subject, and there was no overlap between the samples of each fold. The average classification accuracy (Acc), true positive rate (TPR), and false positive rate (FPR) were calculated to evaluate the performance of the constructed decoding models.

To be specific, the coefficients of kernel function, penalty coefficient C, and other hyperparameters of our SVM were optimized by sequential minimal optimization algorithm for 120 rounds of iterative optimization. The final model used the RBF kernel with *gamma* = 0.05 and penalty coefficient *C* = 1.0. We grouped the training samples again by 5-fold cross validation to determine the hyperparameters of XGBoost and RLDA using grid search. The final optimization results were as follows: the regularization parameter lambda of RLDA was 0.9; XGBoost had *n*_*estimators* = 20, *learning*_*rate* = 0.1, *booster* = ′*dart*′, *max*_*depth* = 10, and default values for the other hyperparameters.

In this study, SVM was implemented by fitcsvm() in MATLAB. XGBoost and RLDA were implemented using python’s Scikit-learn library.

#### Pseudo-online test

To further test the performance of the target detection system, we conducted a pseudo-online test. The performance of the system was evaluated by 3 indicators: system accuracy (SA), false alarm rate (FAR), and hit rate (HR). The false alarm rate was defined as the percentage of target recognition commands that are false alarm by the system when the target does not appear in the process of watching the video. The hit rate was defined as the percentage of all experiments in which the correct hit occurred. The correct hit meant that the system could correctly output the target recognition command within 1,500 ms after the target appeared. System accuracy was a comprehensive indicator of system performance evaluation, which can be calculated by the following equation:

$$\text{System Accuracy}=\frac{\left(1-\text{False Alarm Rate}\right)+\mathrm{Hit}\;\text{Rate}}{2}$$ (3)

For each subject’s data, 80% of the data were randomly selected as the training sample, and the ICA unmixing matrix, PCA compression matrix, and decoding model parameters were trained. The remaining 20% of the experimental data were used as the test sample.

The pseudo-online test took −2 s as the starting point and intercepted samples according to 1-s window width and 100-ms step size. The samples were input into the recognition system in turn, and preprocessed and extracted features according to the pseudo-online testing process. Finally, the classification results were output by the trained classification model.

To avoid false alarms due to the nonstationarity of EEG signals, we set different detection thresholds of 1, 2, and 3, that is, the system will recognize the target only if several consecutive outputs are defined as the target class.

## Results

### Neural signature results

#### ERP results

**Fig. 3.**
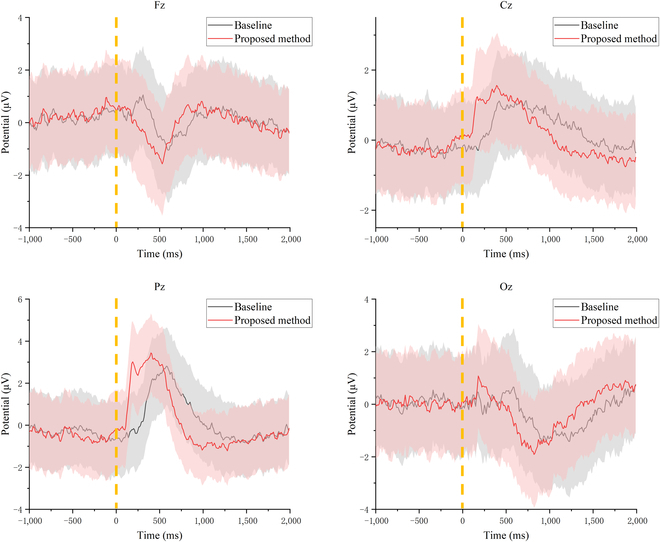
ERPs from Fz, Cz, Pz, and Oz under different epoch extraction methods.

Figure 3 shows the grand average ERPs for all subjects in 4 representative channels Fz, Cz, Pz, and Oz, which contain the results of 2 different epoch extraction methods. The shaded part of the plot line is the standard deviation. The figure showed that the channels were activated, and the ERP response intensity obtained by the proposed epoch extraction method signals was greater, which showed that the method proposed can effectively achieve accurate extraction of ERP segments. Specifically, with our proposed alignment method, a significant positive shift of about 3.4 μV (Pz channel) was observed near the parietal lobe at 300 ms after stimulus onset. The mean amplitude of the 4 representative channels was 0.52 μV higher and the peak appeared 310 ms earlier compared to the baseline. The Wilcoxon test showed that there were significant differences in the peak latency (*P* = 0.0122 < 0.05) of the Pz channel between the 2 epoch extraction methods.

**Fig. 4.**
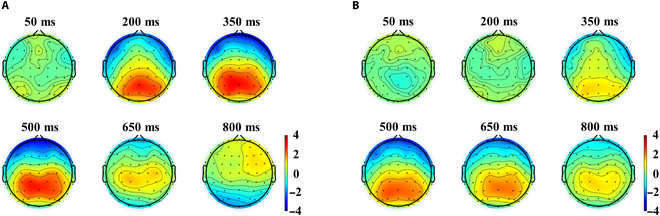
(A) ERP topology (proposed method). (B) ERP topology (Triggerbox-based epoch extraction method).

We also plotted the average brain topology map of all subjects. As shown in Fig. 4, it can be seen that positive waves appeared in the central area, parietal area, and occipital area 200 ms after the alignment time. Furthermore, the response intensity obtained by our proposed method was higher, the response area was larger, and the peak delay was shorter. The greatest response intensity was found in the parietal region of the brain topography, along with a high intensity in the occipital region associated with visual stimuli, which was in line with existing studies [33].

Through the comparison of the above multiple angles, it can be seen that the proposed method effectively achieved ERP alignment, which was more conducive to the training of EEG decoding models.

#### ERSP results

**Fig. 5.**
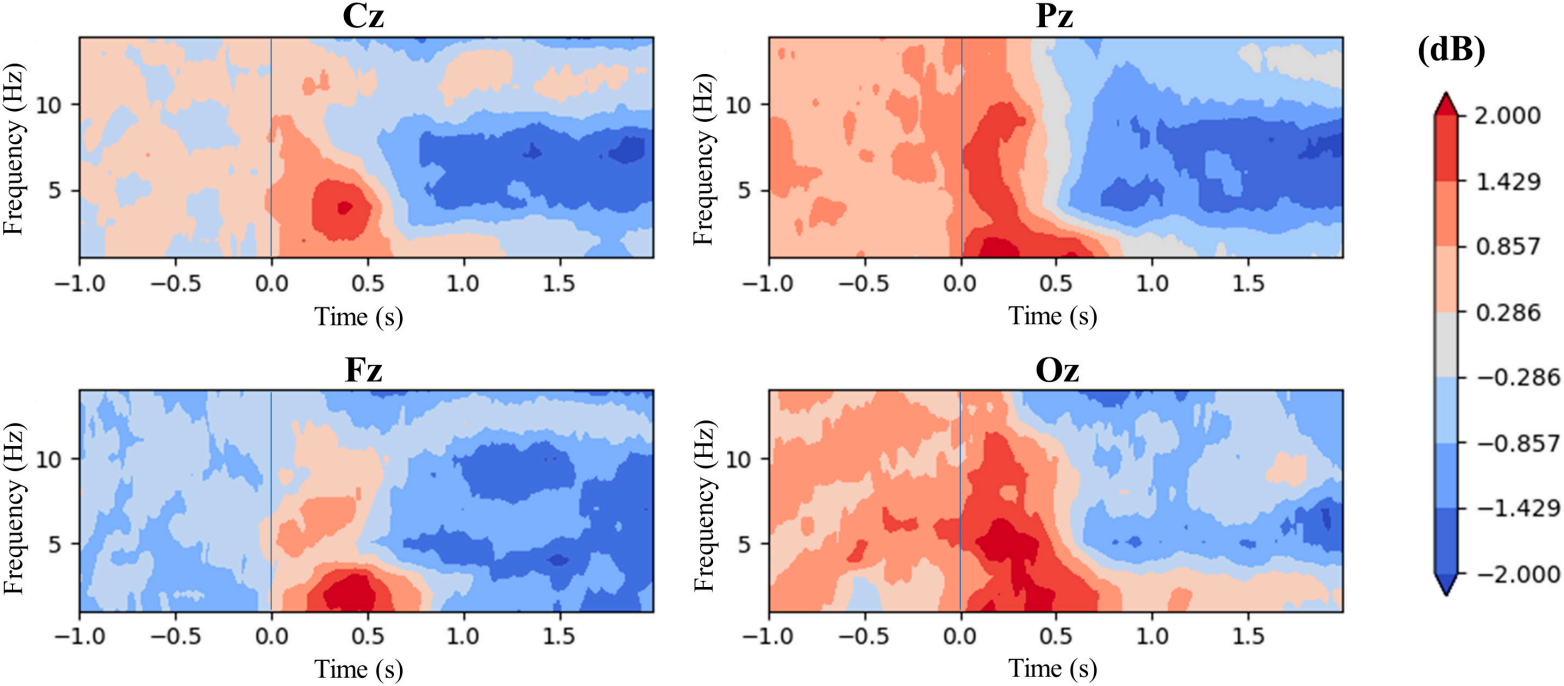
ERSP of EEG signals from central area channels.

Figure 5 shows the time-frequency analysis results of the representative channels, Fz, Cz, Pz, and Oz. Only the results obtained by the proposed method are shown here. In the color chart, red represents positive amplitude, blue represents negative amplitude, and the color depth represents the absolute value of amplitude.

Before and after target recognition, the power of 0.5 to 13 Hz (covering delta, theta, and alpha rhythm) EEG signals in each channel changed significantly. Specifically, 2 significant changes occurred. (a) After target recognition, the delta rhythm power increased significantly, reaching up to 2 dB, and the response time and frequency band varied from channel to channel. (b) There was a significant decrease in alpha and theta rhythm power approximately 700 ms after target recognition. The first change might be related to the concentration of attention at the instant of target recognition, and the second change might be related to the relaxation of attention after target recognition.

#### Source analysis results

**Fig. 6.**
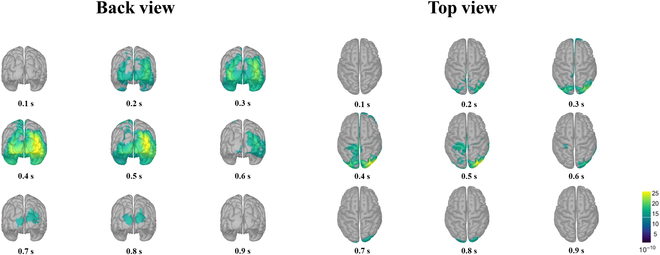
The grand-averaged activation pattern in the source space. The source imaging results are displayed from time lags 0.1 to 0.9 s with the time interval of 0.1 s. The source-space activity was imaged from 2 projection angles: back view and top view.

The results of the source analysis are shown in Fig. 6. It was observed that the source activity in the brain was most obvious within 0 to 800 ms after subjects recognized a target. In this study, the Desikan–Killiany atlas was used to define each brain region and analyze the activity of each brain region [34]. It was observed that the activation occurred first in the lateral occipital lobe about 170 ms after subjects recognized the target. Subsequently, from 180 to 580 ms, the superior parietal lobule also showed persistent activation. From 205 ms, activation appeared in the left and right superior frontal gyri, representing the participation of higher cognition. Activation appeared in the postcentral gyrus around 340 ms and peaked at 510 ms. It should be noted that the lateral occipital lobe, an important part of the human visual pathway, was continuously activated from about 170 to 800 ms.

The results of source analysis showed that detecting targets in the low-quality video activated multiple brain regions of the subjects, providing a neural basis for us to establish the decoding model and target detection system.

### Decoding and detection performance

#### Channel selected for each subject

The channel selection results of all subjects were presented in the supplementary materials (Table S1). Most of the selected channels were concentrated in the central region, occipital lobe, and frontal lobe, which was basically consistent with the previous research on neural representation [35]. For some subjects, the temporal lobe channels were also selected, which might be related to the information integration and recognition function in this region.

#### Off-line classification results

**Fig. 7.**
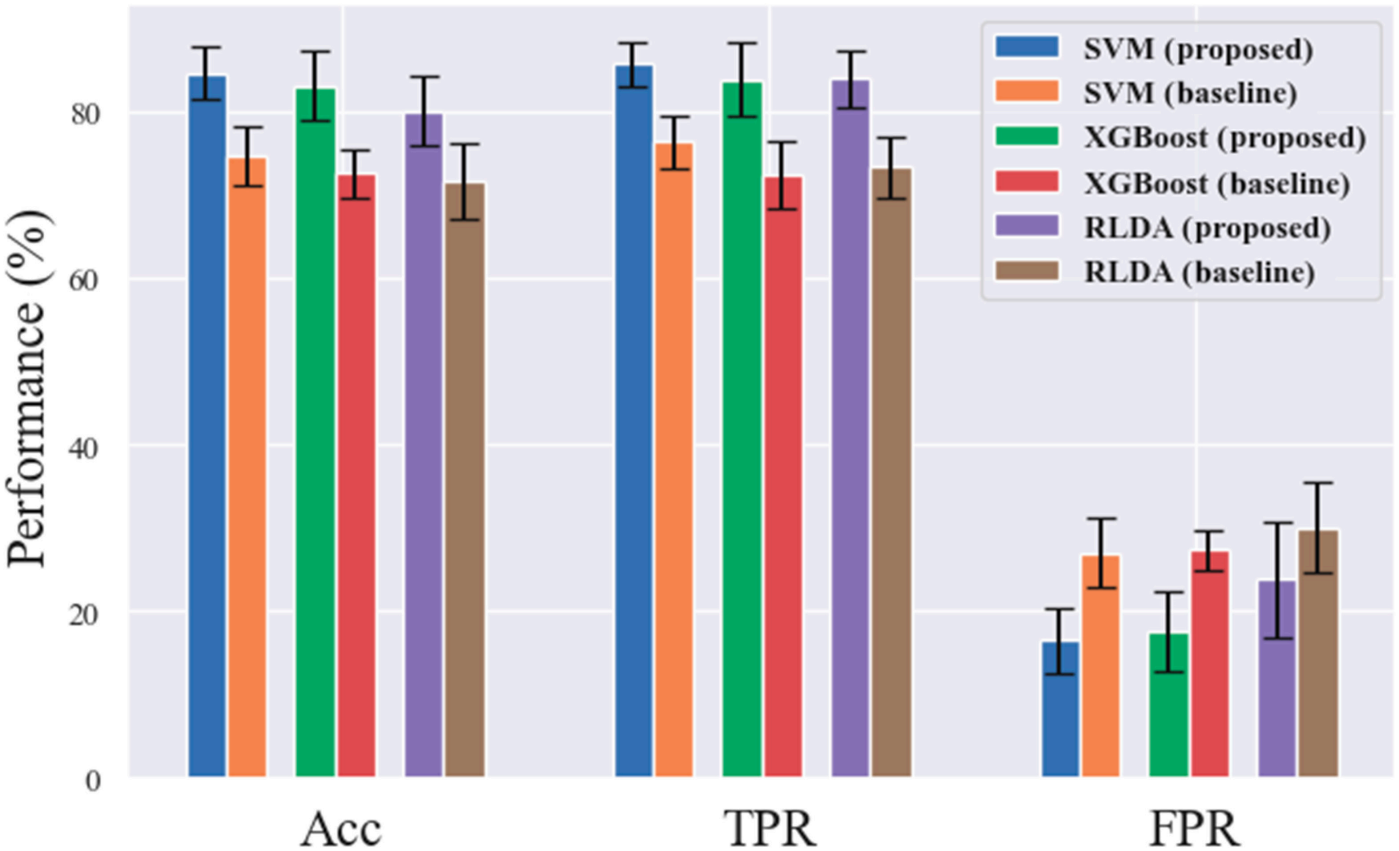
Average results of offline classification of low-quality video target detection for 8 subjects.

The offline classification results of low-quality video target detection for 8 subjects are shown in Fig. 7. In the figure, the Acc, TPR, and FPR of different classifiers are presented. The results of different epoch extraction methods were compared. It was seen that the SVM classifier showed the best performance among the 3 algorithms. The average Acc, TPR, and FPR of the SVM model based on the proposed method were 84.56% ± 3.18%, 85.63% ± 2.65%, and 16.46% ± 3.89%. According to Fig. 7, it was found that the highest Acc of the models based on the baseline method was only 74.64 ± 3.47%. The Wilcoxon test showed that the Acc difference between the 2 methods was significant (*P* = 0.0002 < 0.01), showing that our proposed method can greatly improve the performance and weaken the impact caused by the asynchronous problem. S2 showed the best classification performance with good performance for all 3 metrics. SVM did not always perform best for a particular subject. For example, S5 showed an excellent classification accuracy of 90.42% by the XGBoost algorithm, indicating that, in practice, the classifier should be customized for a certain subject. The RLDA algorithm did not perform well in classification, which might be because the nonlinear dynamic characteristics of EEG make it unsuitable.

#### Pseudo-online detection performance

**Fig. 8.**
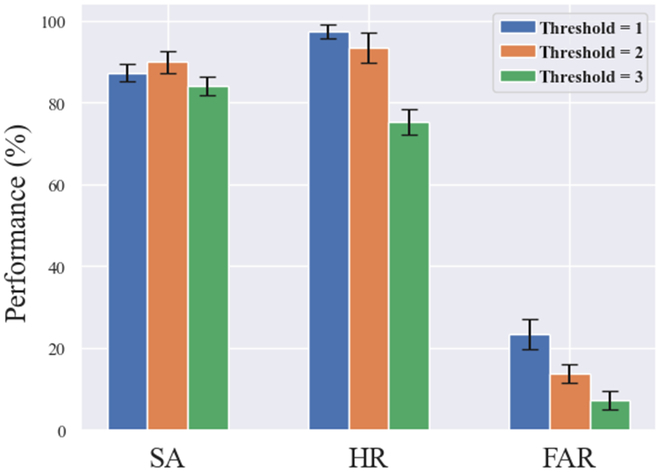
Pseudo-online detection results under different detection thresholds.

In the pseudo-online test, we set 3 detection thresholds. Figure 8 shows the pseudo-online test results based on the 3 detection thresholds. For the 3 detection thresholds, the average HR of the system were 97.39%, 93.43%, and 75.35%, respectively, and the average FAR were 23.34%, 13.74%, and 7.2%, respectively. The SA for the 3 thresholds were 87.2%, 89.84%, and 84.07%. It was seen that, with the increase of the detection threshold, HR and FAR of the system decreased. From the comprehensive index, when the threshold is 2, the SA is the highest, and when the threshold is 1, the HR is the highest. However, the Wilcoxon test showed that there was no significant difference in SA between threshold 1 and threshold 2 (*P* = 0.1242 > 0.05). Since the proportion of nontarget samples was much larger than that of target samples in the process of recognizing video targets (there may be tens of times difference), the impact of FAR on system performance was greater than that of HR, and thus, the threshold value of the system should be set to 2.

## Discussion

In this paper, we investigated low-quality video target detection based on EEG signals. We designed and built an experimental paradigm for low-quality video object recognition by using videos taken by simulated drones during sea surface reconnaissance as stimuli. Unlike previous studies, we did not use the widely used RSVP paradigm [15,16], nor did we use high-quality video targets as our experimental stimuli [17,18]. Instead, low-quality targets with camouflage, mutilation, occlusion, and weakness, which are more common in practice, were simulated as experimental stimuli.

Low-quality target detection based on EEG signals has never been explored in previous research, although it is of great research value. The recognition performance of computer vision for low-quality targets is poor [36]. BCI technology can skillfully combine manual and machine recognition, which may have the high accuracy of manual recognition and the high processing efficiency of machine recognition. Therefore, EEG-based target detection technology has advantages over machine and manual detection.

To address the asynchronous problem in EEG-based video target detection, we proposed an EEG epoch extraction method based on eye movement signals, which used different eye movement types during target recognition to determine the target recognition time and accurately extract the target EEG samples. The effectiveness of the proposed method was verified in the subsequent neural representation analysis and decoding. In recent years, some studies have applied eye movement signals to the field of EEG-based visual object detection. Kaunitz et al. [37] asked subjects to find a hidden target face in a crowded scene with distractor faces while recording eye movement signals and EEG signals to extract ERPs with fixation. Qian et al. [38] used fixation-related potential to command subjects to select target applications from 25 application icons arranged in a 5×5 arrangement. However, the existing research focuses on object detection in images. We aim to solve the problem of asynchronous detection in low-quality video targets and align ERPs more accurately with eye movement signals. This question has not been studied before.

By observing the ERP representation and time-frequency representation of brain activities in the process of low-quality video target detection, we found that the reaction intensity and response range of the average ERP obtained by the proposed epoch extraction method were more extensive than the baseline, which showed that the proposed method could align ERP fragments more accurately. The ERSP analysis showed that the video-target detection caused a decrease in the power of alpha rhythm from the central channel, which indicated a redeployment of subjects’ attention, consistent with existing neuroscience conclusions. Source analysis results showed that video-target detection caused the activation of multiple brain regions, among which the occipital lobe, as an important part of the human visual pathway, was activated initially and continued to be activated later. In addition, the superior frontal gyri, superior parietal lobule, and postcentral gyrus were activated to varying degrees. Based on the time-frequency characteristics of the continuous wavelet transform, we established an EEG recognition model of low-quality video objects and carried out a pseudo-online test. By comparison, we found that the proposed method effectively improved the performance.

In terms of application value, the research can be applied in the military field, such as threat target reconnaissance and disaster relief patrol. In the civilian field, it can be used for surveillance and urban suspicious personnel investigation and tracking. In the medical field, it can help doctors quickly screen target medical images and help reduce the medical process. In addition, this method can extract key information from the video accurately and efficiently.

There are still some limitations in this study, which need to be further explored in the follow-up work. Online experimentation of the low-quality video target detection system is an important issue, and the real-time performance of the whole system needs to be verified. In the practical application process, we still need to pay attention to the impact of pseudo targets on system performance. In the future, we need to compare our method with computer vision or manual methods to more comprehensively evaluate the performance of our proposed method in practical applications, as well as the advantages and disadvantages compared with other methods. In addition, machine intelligence and human intelligence can be fused, and the participation of computer vision can realize a multiclassification target detection system [39,40].

Although low-quality video targets were studied in this study, no quantitative index was proposed for the low-quality degree of targets. In the design of the experiment, we simply simulated the low-quality of the target due to factors such as weather, environment, or the target being partially obscured by clouds, waves, and islands. Although the simulation can reflect the challenge of low-quality video target detection to a certain extent, it is still relatively simple compared with the complex and changeable low-quality situations that may be encountered in the actual scene. In order to better apply the target detection technology based on EEG to the human–computer interaction system, it is necessary to further study the influence of different video object quality parameters (video size, definition, and screen complexity) on target detection.

## Data Availability

All data are available from the corresponding author upon reasonable request.

## References

[1] Zhao N, Lu W, Sheng M, Chen Y, Tang J, Yu FR, Wong K. UAV-assisted emergency networks in disasters. IEEE Wirel Commun. 2019;26(1):45–51.

[2] Miller BM, Stepanyan KV, Popov AK, Miller AB. UAV navigation based on videosequences captured by the onboard video camera. Autom Remote Control. 2017;78(12):2211–2221.

[3] Adão T, Hruška J, Pádua L, Bessa J, Peres E, Morais R, Sousa J. Hyperspectral imaging: A review on UAV-based sensors, data processing and applications for agriculture and forestry. Remote Sens. 2017;9(11):1110.

[4] Gerson AD, Parra LC, Sajda P. Cortical origins of response time variability during rapid discrimination of visual objects. NeuroImage. 2005;28(2):342–353. 16169748 10.1016/j.neuroimage.2005.06.026

[5] Lees S, McCullagh P, Payne P, Maguire L, Lotte F, Coyle D. Speed of rapid serial visual presentation of pictures, numbers and words affects event-related potential-based detection accuracy. IEEE Trans Neural Syst Rehabil Eng. 2020;28(1):113–122. 31751279 10.1109/TNSRE.2019.2953975

[6] Janapati R, Dalal V, Govardhan N, Gupta RS. Review on EEG-BCI classification techniques advancements. IOP Conf Ser Mater Sci Eng. 2020;981(3): Article 032019.

[7] Peng B, Bi L, Wang Z, Feleke AG, Fei W. Robust decoding of upper-limb movement direction under cognitive distraction with invariant patterns in embedding manifold. IEEE Transactions on Neural Systems and Rehabilit Eng. 2024;32:1344–1354. doi:10.1109/tnsre.2024.3379451 10.1109/tnsre.2024.3379451 38502615

[8] Wang J, Bi L, Fei W. Multitask-oriented brain-controlled intelligent vehicle based on Human–Machine Intelligence Integration. IEEE Transactions on Systems, Man, and Cybernetics: Systems. 2023;53(4):2510–2521. doi:10.1109/tsmc.2022.3212744 10.1109/tsmc.2022.3212744

[9] Gerson AD, Parra LC, Sajda P. Cortically coupled computer vision for rapid image search. IEEE Trans Neural Syst Rehabil Eng. 2006;14(2):174–179. 16792287 10.1109/TNSRE.2006.875550

[10] Bigdely-Shamlo N, Vankov A, Ramirez RR, Makeig S. Brain activity-based image classification from rapid serial visual presentation. IEEE Trans Neural Syst Rehabil Eng. 2008;16(5):432–441. 18990647 10.1109/TNSRE.2008.2003381

[11] Hughes G, Mathan S, Yeung N. EEG indices of reward motivation and target detectability in a rapid visual detection task. NeuroImage. 2013;64:590–600. 22982373 10.1016/j.neuroimage.2012.09.003

[12] Wu Q, Zeng Y, Lin Z, Wang X, Yan B. Real-time EEG-based person authentication system using face rapid serial visual presentation. Paper presented at: 2017 8th International IEEE/EMBS Conference on Neural Engineering (NER). 2017 May 25–28; Shanghai China.

[13] Lu R, Zeng Y, Zhang R, Yan B, Tong L. SAST-GCN: Segmentation adaptive spatial temporal-graph convolutional network for P3-based video target detection. Front Neurosci. 2022;16:913027. 35720707 10.3389/fnins.2022.913027 PMC9201684

[14] Rosenthal D, DeGuzman P, Parra LC, Sajda P. Evoked neural responses to events in video. IEEE J Sel Top Signal Process. 2014;8(3):358–365.

[15] Weiden M, Khosla D, Keegan M. Electroencephalographic detection of visual saliency of motion towards a practical brain-computer interface for video analysis. Paper presented at: Proceedings of the 14th ACM international conference on Multimodal interaction. 2012 Oct 22, NY, United States.

[16] Marathe AR, Ries AJ, McDowell K. Sliding HDCA: Single-trial EEG classification to overcome and quantify temporal variability. IEEE Trans Neural Syst Rehabil Eng. 2014;22(2):201–211. 24608681 10.1109/TNSRE.2014.2304884

[17] Mustafa M, Guthe S, Magnor M. Single-trial EEG classification of artifacts in videos. ACM Trans Appl Percept. 2012;9(3):1–15.

[18] Liu X, Tao X, Xu M, Zhan Y, Lu J. An EEG-based study on perception of video distortion under various content motion conditions. IEEE Trans Multimed. 2020;22(4):949–960.

[19] Song X, Yan B, Tong L, Shu J, Zeng Y. Asynchronous video target detection based on single-trial EEG signals. IEEE Trans Neural Syst Rehabil Eng. 2020;28(9):1931–1943. 32746322 10.1109/TNSRE.2020.3009978

[20] Rayner K. Eye movements and cognitive processes in reading, visual search, and scene perception. Stud Visual Info Proces. 1995;6:3–22.

[21] Williams CC. Looking for your keys: The interaction of attention, memory, and eye movements in visual search. Psychology of Learning and Motivation. 2020;73:195–229. doi:10.1016/bs.plm.2020.06.003

[22] Henderson JM, Shinkareva SV, Wang J, Luke SG, Olejarczyk J. Predicting cognitive state from Eye Movements. PLOS ONE. 2013;8(5):e64937. 23734228 10.1371/journal.pone.0064937 PMC3666973

[23] Joseph MacInnes W, Hunt AR, Clarke AD, Dodd MD. A generative model of cognitive state from task and eye movements. Cogn Comput. 2018;10(5):703–717. 10.1007/s12559-018-9558-9 PMC6367733 30740186

[24] Jothi Prabha A, Bhargavi R. Predictive model for dyslexia from fixations and saccadic eye movement events. Comput Methods Prog Biomed. 2020;195:105538. 10.1016/j.cmpb.2020.105538 32526535

[25] Peng H, Li B, He D, Wang J. Identification of fixations, saccades and smooth pursuits based on segmentation and clustering. Intell Data Anal. 2019;23(5):1041–1054.

[26] Winkler I, Debener S, Muller K-R, Tangermann M. On the influence of high-pass filtering on ICA-based artifact reduction in EEG-ERP. Annu Int Conf IEEE Eng Med Biol Soc. 2015;2015:4101–4105. 26737196 10.1109/EMBC.2015.7319296

[27] Hyvärinen A, Oja E. Independent component analysis: Algorithms and applications. Neural Netw. 2000;13(4–5):411–430. 10946390 10.1016/s0893-6080(00)00026-5

[28] Pion-Tonachini L, Kreutz-Delgado K, Makeig S. ICLABEL: An automated electroencephalographic independent component classifier, dataset, and website. NeuroImage. 2019;198:181–197. 31103785 10.1016/j.neuroimage.2019.05.026 PMC6592775

[29] Delorme A, Makeig S. EEGLAB: An open source toolbox for analysis of single-trial EEG dynamics including independent component analysis. J Neurosci Methods. 2004;134(1):9–21. 15102499 10.1016/j.jneumeth.2003.10.009

[30] Makeig S. Auditory event-related dynamics of the EEG spectrum and effects of exposure to tones. Electroencephalogr Clin Neurophysiol. 1993;86(4):283–293. 7682932 10.1016/0013-4694(93)90110-h

[31] Pascual-Marqui RD. Standardized low-resolution brain electromagnetic tomography (sLORETA): technical details. Methods Find Exp Clin Pharmacol. 2002;24(Suppl D):5–12. 12575463

[32] Tadel F, Baillet S, Mosher JC, Pantazis D, Leahy RM. Brainstorm: A user-friendly application for MEG/EEG analysis. Comput Intell Neurosci. 2011;2011:879716. 21584256 10.1155/2011/879716 PMC3090754

[33] Zafar R, Malik AS, Kamel N, Dass SC, Abdullah JM, Reza F, Abul Karim AH. Decoding of visual information from human brain activity: A review of fmri and EEG studies. J Integr Neurosci. 2015;14(2):155–168. 25939499 10.1142/S0219635215500089

[34] Alexander B, Loh WY, Matthews LG, Murray AL, Adamson C, Beare R, Chen J, Kelly C, Anderson PJ, Doyle LW, et al. Desikan-Killiany-Tourville Atlas compatible version of M-crib neonatal parcellated whole brain atlas: The M-CRIB 2.0. Frontiers in Neuroscience. 2019;13:34. 30804737 10.3389/fnins.2019.00034 PMC6371012

[35] Contini EW, Wardle SG, Carlson TA. Decoding the time-course of object recognition in the human brain: From visual features to categorical decisions. Neuropsychologia. 2017;105:165–176. 28215698 10.1016/j.neuropsychologia.2017.02.013

[36] Caiafa CF, Solé-Casals J, Marti-Puig P, Zhe S, Tanaka T. Decomposition methods for machine learning with small, incomplete or noisy datasets. Appl Sci. 2020;10(23):8481.

[37] Kaunitz LN, Kamienkowski JE, Varatharajah A, Sigman M, Quiroga RQ, Ison MJ. Looking for a face in the crowd: Fixation-related potentials in an eye-movement visual search task. NeuroImage. 2014;89:297–305. 24342226 10.1016/j.neuroimage.2013.12.006

[38] Qian L, Ge X, Feng Z, Wang S, Yuan J, Pan Y, Shi H, Xu J, Sun Y. Brain network reorganization during visual search task revealed by a network analysis of fixation-related potential. IEEE Trans Neural Syst Rehabil Eng. 2023;31:1219–1229. 37022804 10.1109/TNSRE.2023.3242771

[39] Robinson RM, Hyungtae Lee, McCourt MJ, Marathe AR, Kwon H, Chau Ton, Nothwang WD. Human-autonomy sensor fusion for rapid object detection. Paper presented at: 2015 IEEE/RSJ International Conference on Intelligent Robots and Systems (IROS). 2015 Sep 28–Oct 02; Hamburg, Germany.

[40] Min J, Cai M, Gou C, Xiong C, Yao X. Fusion of forehead EEG with machine vision for real-time fatigue detection in an automatic processing pipeline. Neural Comput Appl. 2022;23(12):8859–8872.

