# Supplementary material for: Low-Quality Video Target Detection Based on EEG Signal Using Eye Movement Alignment
Source: Cyborg Bionic Syst. 2024 Jul 4;5:0121. doi: 10.34133/cbsystems.0121 (PMC11222288; doi:10.34133/cbsystems.0121)
Supplement: Supplementary 1 — Table S1 [file cbsystems.0121.f1.pdf]

Supplementary Materials

Table S1. Channel selection results for each subject

Subject No.	Selected Channels	Number of Channels
S1	Cz,C3,Pz,P3,P4,Fpz,Fp1,F3,F4,F5,F6,FCz,FC1,FC2,FC3,FC4,FC5,FT8,C1,C2,C5,C6,T7,T8,CP1,CP2,CP3,CP4,TP8,P5, POz,PO3,Oz,O1	34
S2	Cz,C4,Pz,P3, AF3,AF4,AF7,AF8,Fz,F3,F4,F7,F8,FCz,FC1,FC4,FC5,FC6,FT7,FT8,C1,C2,C5,C6,T7,T8,CP1,CP5,CP6, TP8,P5,P6, POz,PO4,PO7,PO8,Oz,O1,O2	39
S3	Cz,C3,C4,Pz,P3,P4,Fp2,AF3,AF4,Fz,F1,F2,F3,F5,F6,F7,F8,FCz,FC1,FC2,FC3,C1,C2,C5,C6,CP4,CP5,CP6,TP7,TP8,P5,P6,P7,P8,POz,PO3,PO4,PO5, PO6,PO7,PO8,Oz,O1,O2	44
S4	Cz,C4,Pz,P3, AF3,AF4,AF7,AF8,Fz,F3,F4,F7,F8,FCz,FC1,FC4,FC5,FC6,FT8,C1,C2,C5,C6,T7,T8,CP1,CP5,CP6, TP8,P5,P6, POz,PO4,PO7,PO8,Oz,O1	37
S5	Cz,C4,Pz,P3,AF8,Fz,F3,F4,F7,F8,FCz,FC4,FC5,FC6, C1,C2,C5,C6,T7,T8,CP1,CP5,CP6, TP8,P5,P6, POz,PO4, O2	29
S6	Cz,C4,Pz,P3, AF3,AF4,AF7,AF8,Fz,F3,F4,F7,F8,FCz,FC1,FC4,FC5,FC6,FT7,FT8,C1,C2,C5,C6,T7,T8,CP1,CP5,CP6, TP8,P5,P6, POz,PO4,PO7,PO8,Oz,O1	38
S7	C4,Pz,P3,Fp1,Fz,F2,F3,F7,FCz,FC1,FC2,FC6,C2,C5,T8,CP1,CP5,TP7,P6,POz,PO3,PO4,PO5,O2	24
S8	Pz,P3,Fp1,AF4,AF7,AF8,Fz,F1,F2,F7,FCz,FC2,FC3,FC6, C1,C6,T7,CP2,CP3,TP7,TP8,P5,P6,P7,PO3,PO4,PO5, PO6,Oz	29
